# Supplementary material for: The protein interactome of the citrus Huanglongbing pathogen Candidatus Liberibacter asiaticus
Source: Nat Commun. 2023 Nov 29;14:7838. doi: 10.1038/s41467-023-43648-7 (PMC10687234; doi:10.1038/s41467-023-43648-7)
Supplement: Supplementary file 1 — Supplementary Information [file 41467_2023_43648_MOESM1_ESM.pdf]

Supplementary Table 1. Previous bacterial interactome studies

Organism	Type of Organism	Total ORFs in Genome	Proteins Interacting in Y2H	% ORF Covered	Total Interactions	Avg. Number PPI per Node	System	Reference
M. loti	gram -	7277	1804	24.8	3121	1.7	ǂGal4	Shimoda et al. (DNA research, 2008)
B. subtilis	gram +	4100	287	7	793	2.8	ǂGal4	Marchadier et al. (Proteomics, 2011)
Synechocystis sp.	gram -	3692	1920	52	3236	1.7	Gal4	Sato et al. (DNA research, 2007)
E. coli	gram -	3600	1269	35.3	2234	1.8	Gal4	Rajagopala et al. (Nature biotechnology, 2014)
S. pneumoniae	gram +	2109	820	38.9	2045	2.5	LexA	Wuchtly et al. (ASM molecular biology and physiology, 2017)
C. jejuni	gram -	1654	1321	79.9	2884	2.2	LexA	Parrish et al. (Genome Biology, 2007)
1st *H. pylori*	gram -	1587	261	16.4	908	3.5	Gal4	Rain et al. (Nature, 2001)
2nd *H. pylori*	gram -	1587	739	46.6	1515	2.1	Gal4	Hausert et al. (Molecular and Cellular Proteomics, 2014)
"Comprehensive" *H. pylori*	gram -	1587	1135	71.5	2369	2.1	Gal4	Rain & Hausert et al. (Combined Study)
*T. pallidum**	gram -	1039	726	69.9	3649	5	Gal4	Titz et al. (PLOS One, 2008)
CLas*	gram -	1027	542	52.8	4245	7.8	Gal4	This Study
M. pneumoniae	gram +	689	617	90	10083	16.34	TAP-MS	Kuhner et al. (Science, 2009)

*Obligate intracellular; ǂ: Not a complete interactome.

Supplementary Table 2. 542 CLas proteins were queried for their predicted interactions available from the STRING-db

	STRING-db 0.4[a]		STRING-db 0.9[b]	
	nodes	edges	nodes	edges
All STRING association types	436	3509	256	863
Experimentally determined	115	474	81	369

Note: Two STRING datasets of experimentally proven PPIs (interaction confidence scores of ≥0.4[a] and ≥0.9[b]). The resulting network node and edge counts are listed in the table. No lab experiment datasets determined for CLas are available from STRING. All interaction associations are predicted based on experiments conducted in orthologs.

Supplementary Table 3. PPIs between 22 flagellar proteins in the CLas Y2H

**Interaction type**	**CLas_whole Node**	**CLas_whole edge**	**CLas_HC node**	**CLas_HC edge**
annotated Flg proteins – all PPIs	213	399	118	175
annotated Flg proteins – annotated protein	122	240	59	103
annotated Flg proteins – uncharacterized	69	151	40	67
annotated Flg proteins – annotated Flg proteins	9	8	7	5

Supplementary Table 4. CLas PPIs involving flagellar and membrane proteins. **denotes hypothetical proteins and their annotation given in this study

Protein names	Common Name	Interacting proteins	Common Name	Interaction
CLIBASIA_RS01145	MarR	CLIBASIA_RS00005	*na*	Y2H
CLIBASIA_RS01945	SecB	CLIBASIA_RS00005	*na*	confirmed by pull down
CLIBASIA_RS01945	SecB	CLIBASIA_RS00005	*na*	Y2H
CLIBASIA_RS03390	*na*	CLIBASIA_RS00005	*na*	confirmed by pull down
CLIBASIA_RS03390	*na*	CLIBASIA_RS00005	*na*	Y2H
CLIBASIA_RS01945	SecB	CLIBASIA_RS03165	*na*	Y2H
CLIBASIA_RS03290	TolB	CLIBASIA_RS03395	*na*	Y2H
CLIBASIA_RS01290	FlgB	CLIBASIA_RS03890	*na*	confirmed by pull down
CLIBASIA_RS01290	FlgB	CLIBASIA_RS03890	*na*	Y2H
CLIBASIA_RS01290	FlgB	CLIBASIA_RS05050	*na*	confirmed by pull down
CLIBASIA_RS01290	FlgB	CLIBASIA_RS05050	*na*	Y2H
CLIBASIA_RS01945	SecB	CLIBASIA_RS05050	*na*	Y2H
CLIBASIA_RS03395	FliK**	CLIBASIA_RS05245	BamD	Y2H
CLIBASIA_RS04925	MrcA	CLIBASIA_RS01685	FabA	Y2H
CLIBASIA_RS05260	FtsA	CLIBASIA_RS01685	FabA	Y2H
CLIBASIA_RS03410	FlgK	CLIBASIA_RS03430	FlgD	Y2H
CLIBASIA_RS01255	FlgH	CLIBASIA_RS01275	FlgG	Y2H
CLIBASIA_RS01945	SecB	CLIBASIA_RS03410	FlgK	confirmed by pull down
CLIBASIA_RS03415	FlgL	CLIBASIA_RS03410	FlgK	confirmed by pull down
CLIBASIA_RS03480	FlgN**	CLIBASIA_RS03410	FlgK	Y2H
CLIBASIA_RS03480	FlgN**	CLIBASIA_RS03415	FlgL	Y2H
CLIBASIA_RS01230	EtfA	CLIBASIA_RS01280	FliE	confirmed by pull down
CLIBASIA_RS01230	EtfA	CLIBASIA_RS01280	FliE	Y2H
CLIBASIA_RS01255	FlgH	CLIBASIA_RS01280	FliE	Y2H
CLIBASIA_RS01290	FlgB	CLIBASIA_RS01280	FliE	Y2H
CLIBASIA_RS02290	MrcA	CLIBASIA_RS04130	RlpA	Y2H
CLIBASIA_RS05260	FtsA	CLIBASIA_RS04130	RlpA	Y2H
CLIBASIA_RS03410	FlgK	CLIBASIA_RS03480	*na*	confirmed by pull down
CLIBASIA_RS03410	FlgK	CLIBASIA_RS03915	*na*	Y2H
CLIBASIA_RS04130	RlpA	CLIBASIA_RS00450	*na*	Y2H
CLIBASIA_RS04900	FtsW	CLIBASIA_RS00450	*na*	Y2H

CLIBASIA_RS04900	FtsW	CLIBASIA_RS03165	*na*	Y2H
CLIBASIA_RS04900	FtsW	CLIBASIA_RS03915	*na*	Y2H
CLIBASIA_RS04900	FtsW	CLIBASIA_RS05515	*na*	Y2H
CLIBASIA_RS04960	VWA-domain	CLIBASIA_RS00450	*na*	Y2H
CLIBASIA_RS05050	*na*	CLIBASIA_RS05465	*na*	confirmed by pull down
CLIBASIA_RS05265	FtsQ	CLIBASIA_RS02370	*na*	Y2H
CLIBASIA_RS05465	*na*	CLIBASIA_RS03395	*na*	Y2H
CLIBASIA_RS05515	*na*	CLIBASIA_RS00005	*na*	Y2H

Supplementary Table 5. CLas protein-protein interactions that were confirmed by pull-down assay

**CLas Locus id**	**CLas protein molecular weight (kDa)**	**protein + GST VECTOR (kDa)**	**protein + MBP VECTOR (kDa)**	**NCBI Annotation**
CLIBASIA_RS00005	14.4	40.4	56.4	hypothetical protein
CLIBASIA_RS01230	34.5	60.5	na	FixB
CLIBASIA_RS01280	11.7	na	53.7	FliE
CLIBASIA_RS01290	14.6	Na	56.6	FlgB
CLIBASIA_RS01945	17.4	43.4	59.4	SecB
CLIBASIA_RS03390	45.8	Na	87.8	chemotaxis protein
CLIBASIA_RS03410	53.9	79.9	na	FlgK
CLIBASIA_RS03415	40.2	Na	82.2	FlgL
CLIBASIA_RS03480	13.9	Na	55.9	hypothetical protein (putative FlgN)
CLIBASIA_RS03890	9.4	35.4	na	hypothetical protein
CLIBASIA_RS05050	11.2	37.2	na	hypothetical protein
CLIBASIA_RS05465	7.4	Na	49.4	hypothetical protein

A)

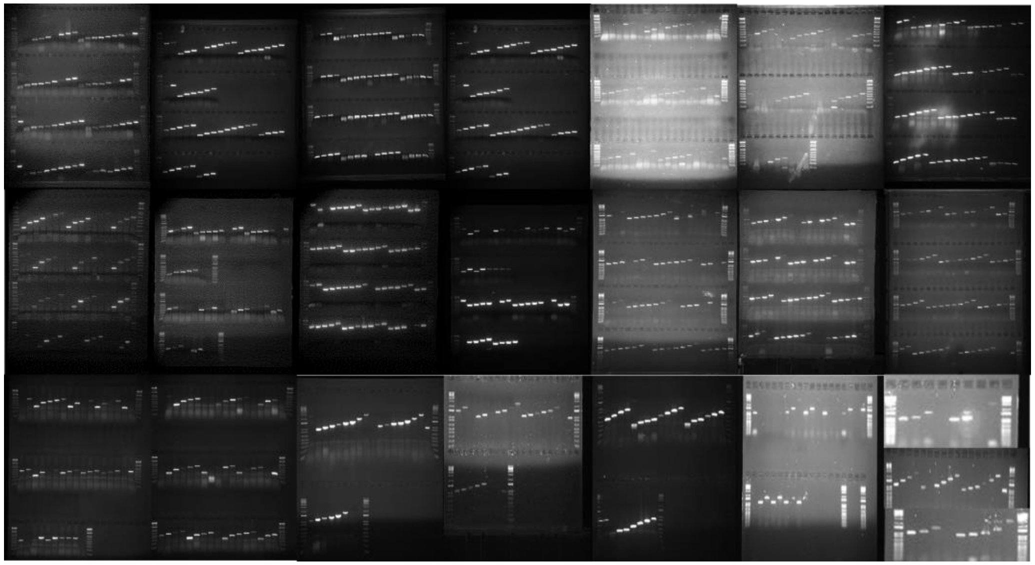

B)

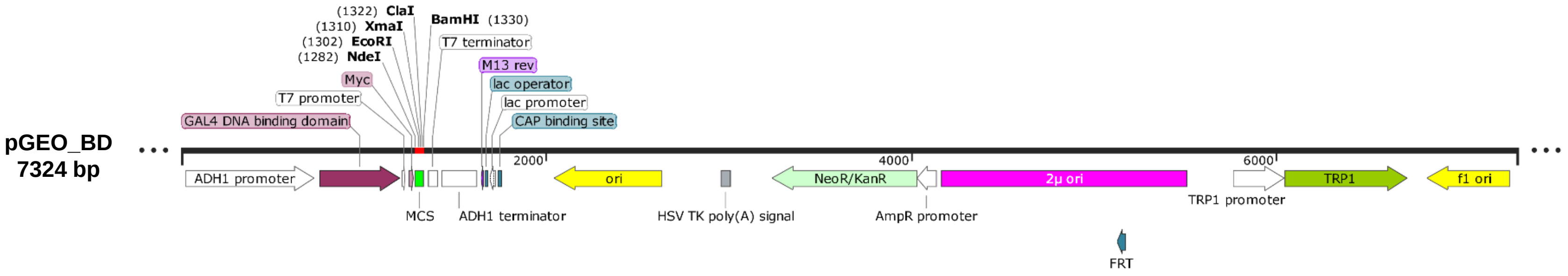

Supplementary Figure 1. Cloning of CLas genes for Y2H. A) Amplification of CLas ORFs for infusion cloning. Primers for 1027 CLas ORFs were designed, resulting in 974 successfully amplified genes for Y2H high throughput interaction screening. **B) Modification of Yeast Two-Hybrid expression vectors enables efficient cloning of all CLas ORFs into two vectors.** A fragment containing the pGADT7 AD (clonetech) MCS sequence between NdeI and BamHI was used to replace the MCS in pGBKT7 (clonetech) between the restriction sites NdeI and BamHI to create a new binding domain vector pGEO_BD. The modified MCS allowed us to design only one primer set for each CLas gene to clone into both AD and BD vectors using Infusion cloning (Clonetech) for the Y2H high-throughput screening. MCS: multiple cloning site.

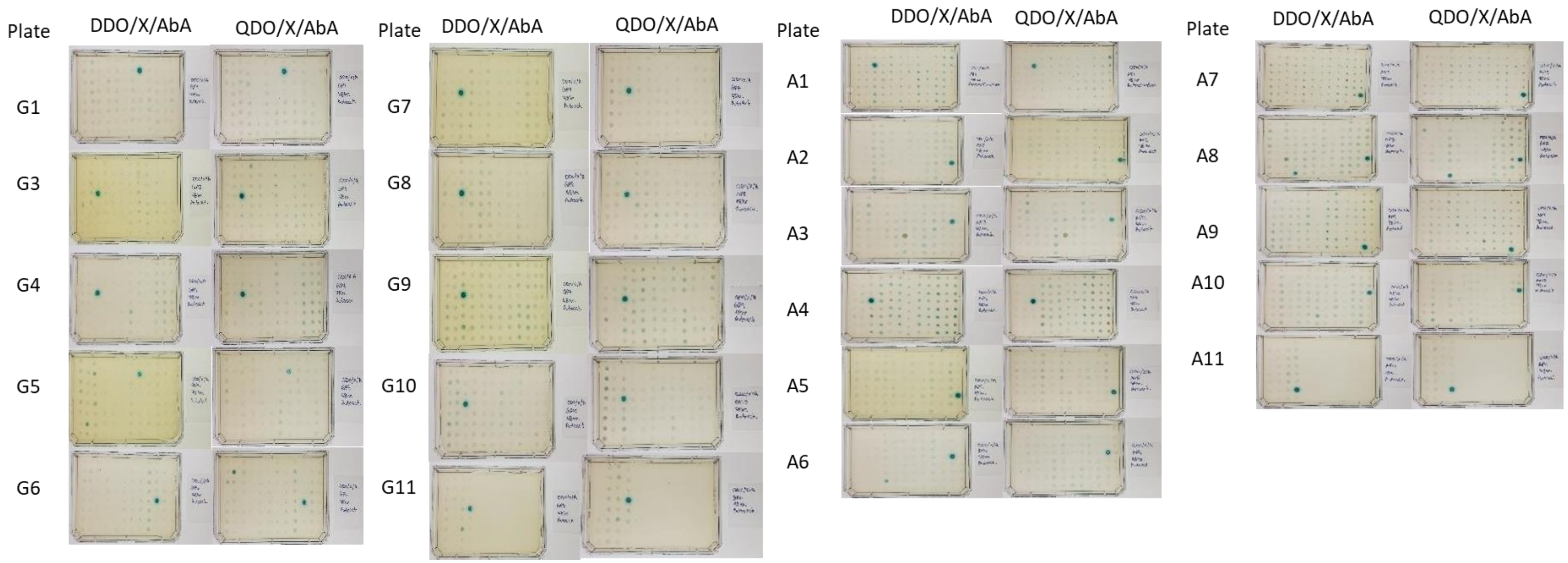

Supplementary Figure 2. Constructing and testing yeast expression vectors. Autoactivation screening for CLas ORF constructs in both AD and BD vectors. From the ORFs cloned into both the AD and BD Y2H vectors, constructs were transformed into Mat-a yeast and mated with Mat-α yeast harboring the empty vector opposite of the construct; i.e., AD construct mated with BD empty vector, vice versa.

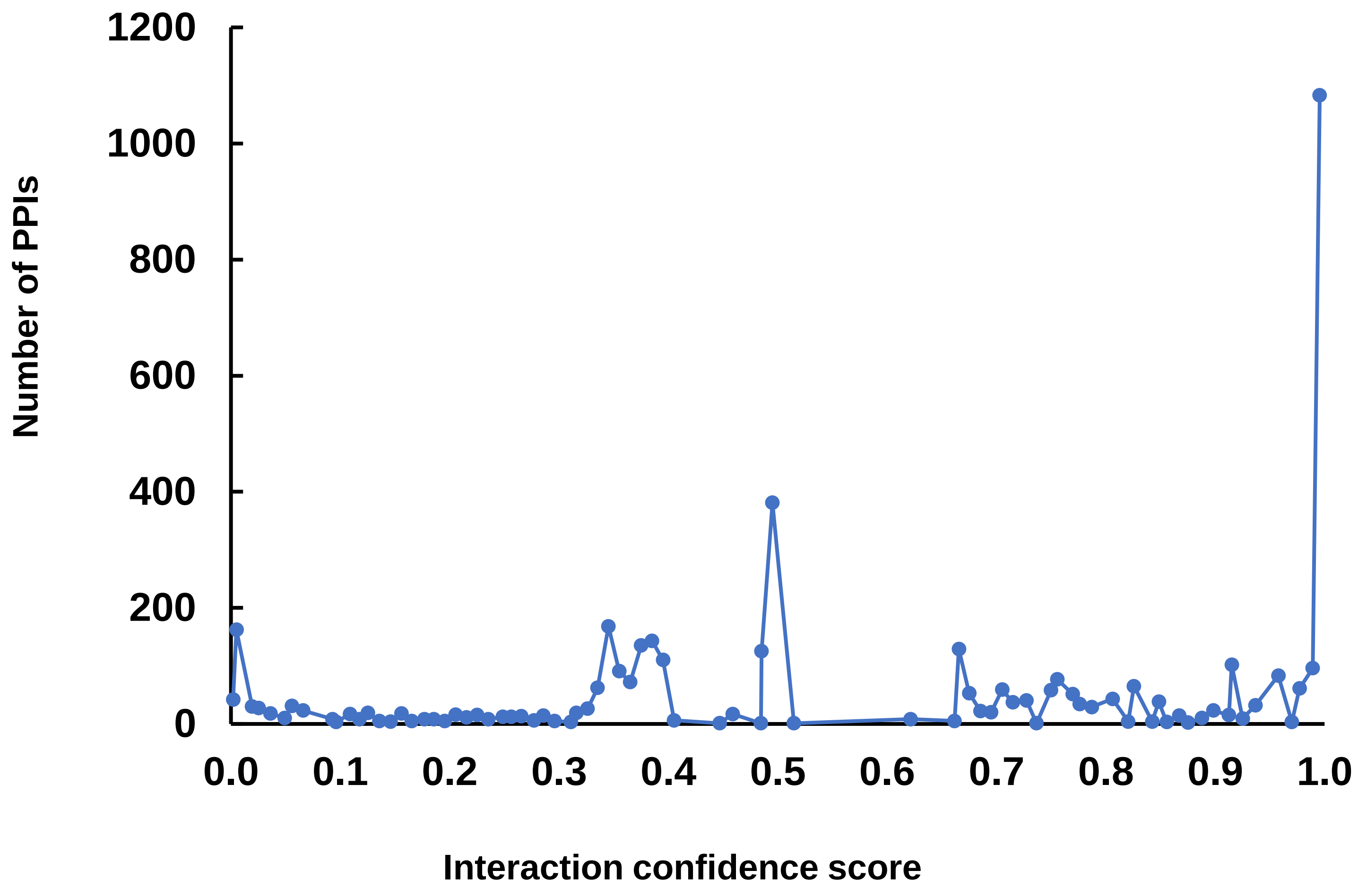

Supplementary Figure 3. Interaction confidence score frequency across the CLas_whole network. There are a total of 4245 PPIs.

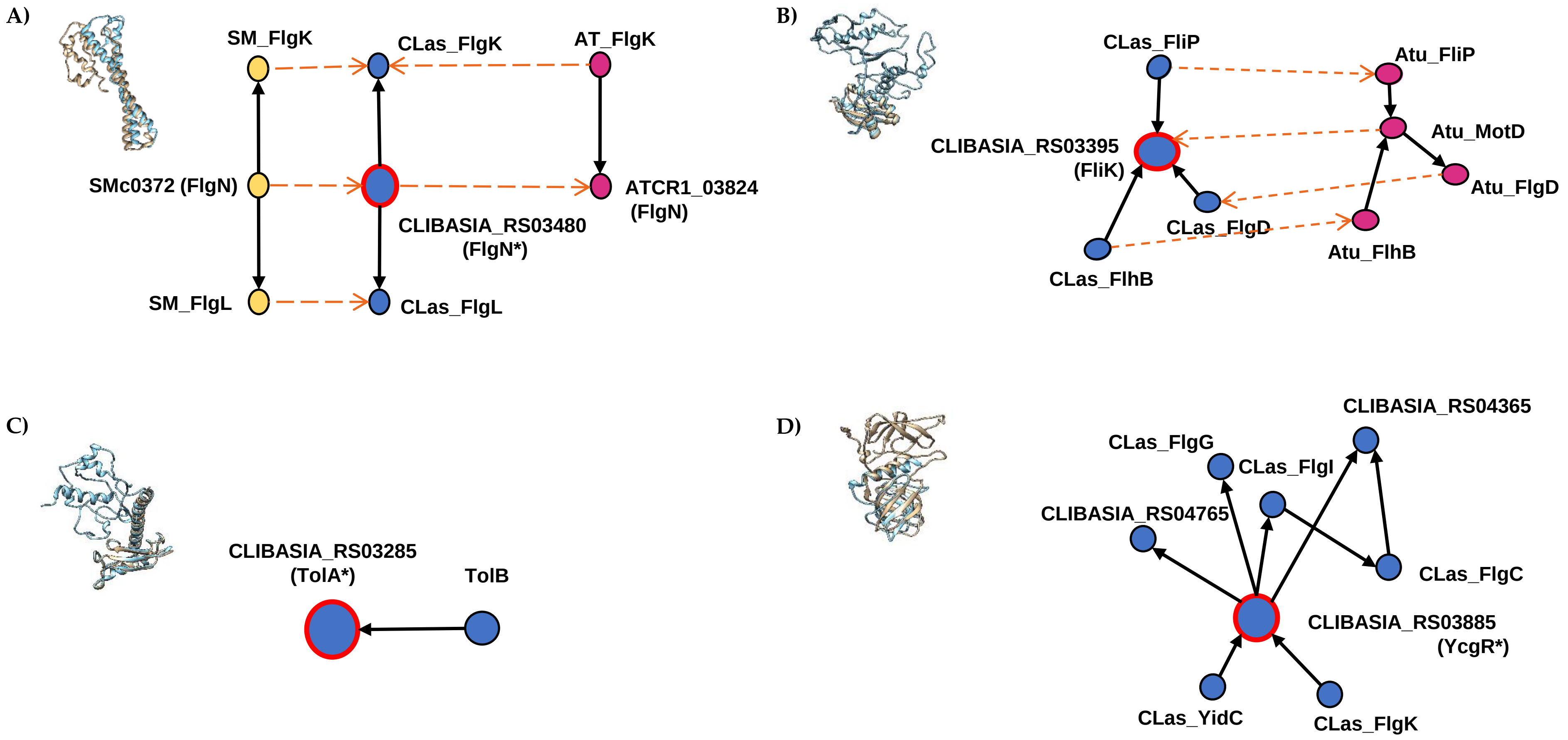

	Locus ID	CLas uncharacterized protein NCBI annotation	annotation this study	structure identity (%)	confidence score (100)	AA sequence identity (%)	AA query cover (%)
A	CLIBASIA_RS03480	hypothetical protein	FlgN	31	83.2	17.89	79
B	CLIBASIA_RS03395	hypothetical protein; putative MotD	FliK	68.83	90.2	36.11	18
C	CLIBASIA_RS03285	hypothetical protein	TolA	45.63	100	35.05	35
D	CLIBASIA_RS03885	PilZ domain-containing protein	YcgR	25	98	25.93	11

Supplementary Figure 4. Protein-protein interactions, interologs, and protein structure homology data were used to assign putative functions for CLas uncharacterized proteins. The Phyre2 online database was used to predict CLas protein structures and find possible template matches of HMM homolog proteins and characterized proteins. Protein structure alignments of CLas proteins (blue), aligned and superimposed over protein Phyre2 protein templates (gold). Alignment and confidence data for each are shown below the figures**. A)** Protein structure alignment of CLIBASIA_RS03480 and *Bradyrhizobium sp.* FlgN. The interologs in *S. meliloti* (yellow) and *A. tumefaciens* (purple) suggest that CLIBASIA_RS03480 To be FlgN. **B)** CLIBASIA_RS3395 was annotated to be FliK based on structure alignment and interologs in *A. bacterium*. **C)** CLIBASIA_RS3285 was annotated to be TolA based on structure alignment and its interaction with CLas TolB in the Y2H network. **D)** CLIBASIA_RS03885 was annotated to be YcgR flagellar brake protein based on structure alignment and interacting partners in Y2H. * annotation was conducted in this study.

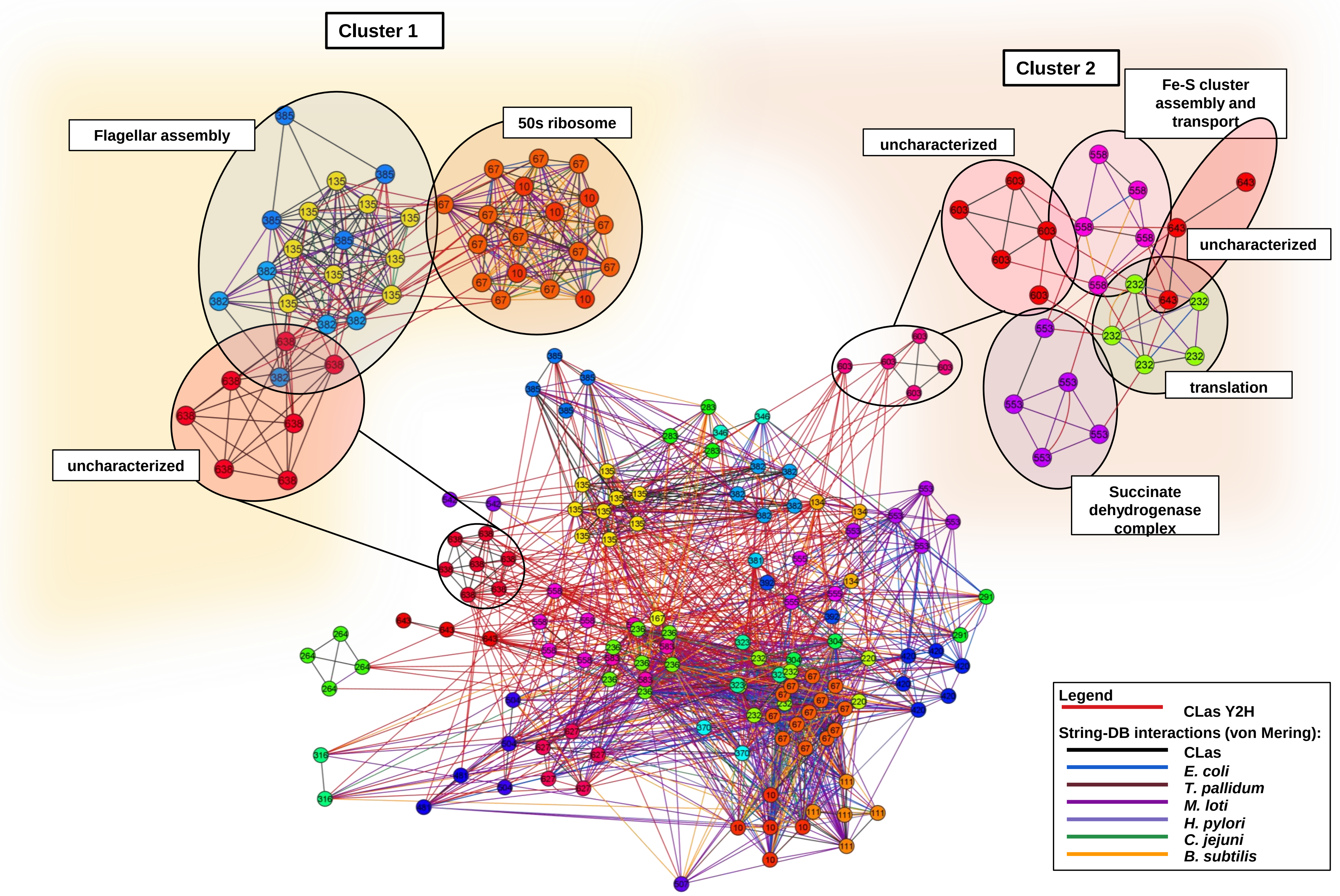

Supplementary Figure 5. Inter-operon interactions highlight associations between motility related genes, respiration, membrane integrity, ribosomal proteins, and uncharacterized genes and operons. The large network shown below the two subnetwork clusters is the CLas operon network. Operons were predicted using the ProOpDB: Prokaryotic Operon Data Base (http://operons.ibt.unam.mx/OperonPredictor). CLas operons with ≥2 PPIs with another operon are shown. Three operonic gene clusters of uncharacterized proteins interact with annotated CLas proteins; uncharacterized operons are in red; i.e.: 603, 638, and 643. Edges are colored by PPI source. The CLas Y2H PPIs in this study are represented by red edges.

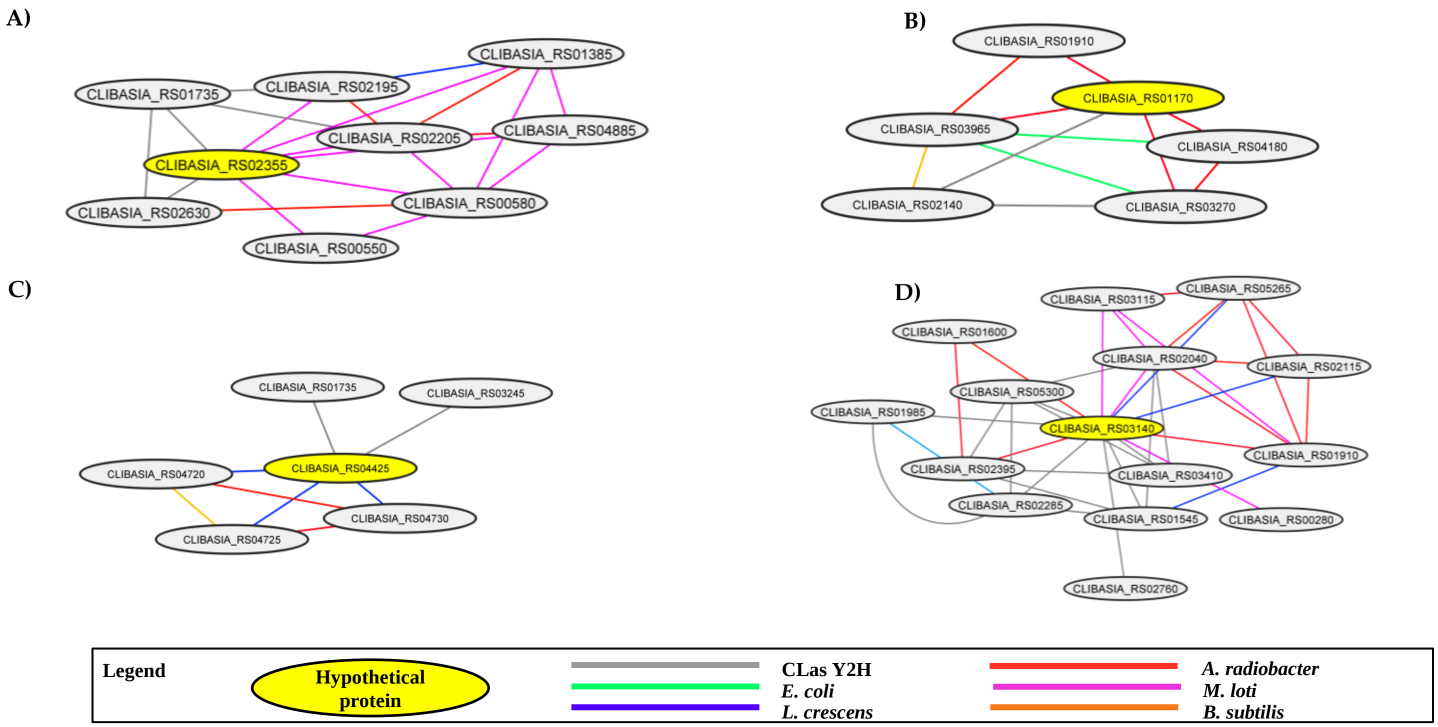

Supplementary Figure 6. Meta-interactome subnetworks. Four hypothetical proteins whose protein function was inferred by its ortholog associations found in meta-interactome (mY2H) subnetworks. Edges are colored by ortholog PPI. A. CLIBASIA_RS02355 has a putative association with cell wall formation and lipid synthesis. B. CLIBASIA_RS04425 has a putative association with FeS cluster assembly, DNA YacG, and RNA pyro phosphohydrolase. C. CLIBASIA_RS0170 has a putative association with DNA processing related proteins. D. CLIBASIA_RS03140 has a putative association with cell division.
