# Supplementary material for: The protein interactome of the citrus Huanglongbing pathogen Candidatus Liberibacter asiaticus
Source: Nat Commun. 2023 Nov 29;14:7838. doi: 10.1038/s41467-023-43648-7 (PMC10687234; doi:10.1038/s41467-023-43648-7)
Supplement: Supplementary file 3 — Description of Additional Supplementary Files [file 41467_2023_43648_MOESM3_ESM.pdf]

Description of Additional Supplementary Files

Supplementary Data 1. CLas primers for Y2H infusion vector construction

Supplementary Data 2. Complete list of binary interactions with NCBI annotations, and interactions score (Scores are 0 to 1; 0.5 to 1 are considered high confidence.)

Supplementary Data 3. Uncharacterized proteins identified in the CLas Y2H interactome

Supplementary Data 4. 163 CLas proteins screened one-to-one for validating the CLas 3-phase screening.

Supplementary Data 5. Distribution of interolog source for each protein pair. Interactions were downloaded from the STRING-db for A. radiobacter, B. subtilis, C. jejuni, E. coli, H. pylori, M. genitallium, M. pneumoniae, S. cerevisiae, S. meliloti, M. loti, T. pallidum, predicted L. crescens, predicted CLas, and COG associations were queried against the CLas_whole network. Reciprocal interactions were removed for simplicity.

Supplementary Data 6. Interologs in the CLas Y2H network

Supplementary Data 7. List of high confidence binary interactions with NCBI annotations, and interactions score

Supplementary Data 8. Forty HUB nodes identified using all three methods: manual curation (DBc), MCC, and MCODE; high confidence HUBs are in red text. (transcriptome data: De Francesco et al., 2022)

Supplementary Data 9. Intra- and inter-operon interactions in the CLas Y2H. STRING interactions from B. subtilis, E. coli, C. jejuni, H. pylori, M. loti, T. pallidum, and predicted CLas (source labeled as STRING) were compared to the CLas Y2H data. PPIs with interologs in the CLas Y2H are in red text.

Supplementary Data 10. CLas operons were predicted using the Prokaryotic Operon DataBase (ProOpDB, http:// operons.ibt.unam.mx/ OperonPredictor) Predicted operons and CLas protein ID assignment is in table A, the gene pair probability table provided by ProOpDB is in table B

Supplementary Data 11. CLas uncharcterized proteins and their interactors for PPI guilt-by-association analysis

Supplementary Data 12. CLas hypothetical proteins given putative functions based on guilt by associations(GBA). Legend: Sec-dependent effector (SDE); Operon and assigned number (O_#)

Supplementary Data 13. 4037 novel CLas PPIs identified using Y2H

Supplementary Data 14. Novel PPIs between annotated proteins of CLas

Supplementary Data 15. Flagellar PPIs in the CLas Y2H network

Supplementary Data 16. CLas gene-protein information including Uniprot IDs and annotations
