# Supplementary material for: The protein interactome of the citrus Huanglongbing pathogen Candidatus Liberibacter asiaticus
Source: Nat Commun. 2023 Nov 29;14:7838. doi: 10.1038/s41467-023-43648-7 (PMC10687234; doi:10.1038/s41467-023-43648-7)
Supplement: Supplementary file 5 — Reporting Summary [file 41467_2023_43648_MOESM5_ESM.pdf]

nature portfolio

Corresponding author(s): Nian Wang

Last updated by author(s): Nov 4, 2023

Reporting Summary

Nature Portfolio wishes to improve the reproducibility of the work that we publish. This form provides structure for consistency and transparency in reporting. For further information on Nature Portfolio policies, see our Editorial Policies and the Editorial Policy Checklist.

Statistics

For all statistical analyses, confirm that the following items are present in the figure legend, table legend, main text, or Methods section.

n/a	Confirmed	
☐	☒	The exact sample size (*n*) for each experimental group/condition, given as a discrete number and unit of measurement
☒	☐	A statement on whether measurements were taken from distinct samples or whether the same sample was measured repeatedly
☒	☐	The statistical test(s) used AND whether they are one- or two-sided<br>*Only common tests should be described solely by name; describe more complex techniques in the Methods section.*
☒	☐	A description of all covariates tested
☒	☐	A description of any assumptions or corrections, such as tests of normality and adjustment for multiple comparisons
☐	☒	A full description of the statistical parameters including central tendency (e.g. means) or other basic estimates (e.g. regression coefficient) AND variation (e.g. standard deviation) or associated estimates of uncertainty (e.g. confidence intervals)
☒	☐	For null hypothesis testing, the test statistic (e.g. *F*, *t*, *r*) with confidence intervals, effect sizes, degrees of freedom and *P* value noted<br>*Give P values as exact values whenever suitable.*
☒	☐	For Bayesian analysis, information on the choice of priors and Markov chain Monte Carlo settings
☒	☐	For hierarchical and complex designs, identification of the appropriate level for tests and full reporting of outcomes
☒	☐	Estimates of effect sizes (e.g. Cohen's *d*, Pearson's *r*), indicating how they were calculated

Our web collection on statistics for biologists contains articles on many of the points above.

Software and code

Policy information about availability of computer code

Data collection	Cytoscape network software, NetworkRandomizer version 3, NCBI database, IMG/MER database, STRING 12.0, DEG database
Data analysis	Cytoscape network software, NetworkRandomizer version 3,NCBI database, IMG/MER database, STRING 12.0, DEG databaseMCODE clustering, and MCC algorithm

For manuscripts utilizing custom algorithms or software that are central to the research but not yet described in published literature, software must be made available to editors and reviewers. We strongly encourage code deposition in a community repository (e.g. GitHub). See the Nature Portfolio guidelines for submitting code & software for further information.

Data

Policy information about availability of data

All manuscripts must include a data availability statement. This statement should provide the following information, where applicable:

- Accession codes, unique identifiers, or web links for publicly available datasets
- A description of any restrictions on data availability
- For clinical datasets or third party data, please ensure that the statement adheres to our policy

The datasets generated and/or analysed during the current study are available in the Supplementary Information, Supplementary Data and Source Data files. Source data are provided with this paper.

Research involving human participants, their data, or biological material

Policy information about studies with human participants or human data. See also policy information about sex, gender (identity/presentation), and sexual orientation and race, ethnicity and racism.

Reporting on sex and gender	Not applicable.
Reporting on race, ethnicity, or other socially relevant groupings	Not applicable.
Population characteristics	Not applicable.
Recruitment	Not applicable.
Ethics oversight	Not applicable.

Note that full information on the approval of the study protocol must also be provided in the manuscript.

Field-specific reporting

Please select the one below that is the best fit for your research. If you are not sure, read the appropriate sections before making your selection.

☒ Life sciences ☐ Behavioural & social sciences ☐ Ecological, evolutionary & environmental sciences

For a reference copy of the document with all sections, see nature.com/documents/nr-reporting-summary-flat.pdf

Life sciences study design

All studies must disclose on these points even when the disclosure is negative.

Sample size	No statistical methods were used to predetermine sample size. We have followed the experimental design of similar studies on Y2H such as Shimoda et al. (DNA research, 2008); Marchadier et al. (Proteomics, 2011); Sato et al. (DNA research, 2007); Rajagopala et al.(Nature biotechnology, 2014); Wuchtly et al. (ASM molecular biology and physiology, 2017)
Data exclusions	No
Replication	For the pull-down assays, each experiment was repeated independently at least twice with similar results. Source data are provided as a Source data file. For the Y2H assays, proteins interactions were conducted on multiple medida including the most stringent media, QX (-trp, -leu, -ade, -his, +XαGal) and QXA (-trp, -leu, -ade, -his, +XαGal, +AbA), which are considered as independent replicates. We further confirmed the data by pairwise rescreening.
Randomization	Because it is a genome wide study, the arrangement of the constructs in the 96-well plate is indeed random.
Blinding	Because it is a genome wide study, it can be considered as a blinding test because we are not aware which construct is in which well of the 96-well plate.

Reporting for specific materials, systems and methods

We require information from authors about some types of materials, experimental systems and methods used in many studies. Here, indicate whether each material, system or method listed is relevant to your study. If you are not sure if a list item applies to your research, read the appropriate section before selecting a response.

Materials & experimental systems

n/a	Involved in the study	
☐	☒	Antibodies
☒	☐	Eukaryotic cell lines
☒	☐	Palaeontology and archaeology
☒	☐	Animals and other organisms
☒	☐	Clinical data
☒	☐	Dual use research of concern
☒	☐	Plants

Methods

n/a	Involved in the study	
☒	☐	ChIP-seq
☒	☐	Flow cytometry
☒	☐	MRI-based neuroimaging

Antibodies

Antibodies used	anti-GST (ab92, Abcam, 1:1,000 dilution), anti-MBP (E8032S, NEB, 1:10,000 dilution), and Goat Anti-Mouse IgG H&L (HRP) (ab205719, Abcam, 1:10,000)
Validation	Validation can be found in Product Specification Sheet, Certificate of Analysis, data card or product manual of NEB, and Abcam.

Plants

Seed stocks	Not applicable.
Novel plant genotypes	Not applicable.
Authentication	Not applicable.
